# Left-Sided Pure Trigeminal Motor Neuropathy With Radiologically Isolated Contralateral Subclinical Involvement: A Case Report

**DOI:** 10.7759/cureus.108718

**Published:** 2026-05-12

**Authors:** Crazy Mandadi, Vinutha Honneshaiah, Pavan B Sanjeevappa

**Affiliations:** 1 Radiology, Sri Siddhartha Academy of Higher Education, Tumkur, IND

**Keywords:** cranial neuropathy, facial asymmetry, magnetic resonance imaging, masticatory muscle atrophy, pure trigeminal motor neuropathy, trigeminal nerve

## Abstract

A 54-year-old woman presented with slowly progressive left facial asymmetry and jaw weakness over three years, without sensory disturbances or other cranial nerve deficits. Clinical examination revealed wasting of the left-sided muscles of mastication with preserved facial sensation. Magnetic resonance imaging demonstrated marked fatty atrophy involving the left temporalis, masseter, medial, and lateral pterygoid muscles, along with subtle contralateral fatty infiltration of the right medial pterygoid muscle, suggesting possible early subclinical involvement. Fat-suppressed and high-resolution MRI sequences demonstrated no active muscle edema, skull base lesion, or abnormality involving the cisternal segments of the trigeminal nerves. Electromyography supported chronic neurogenic denervation involving the left-sided muscles of mastication. Pure trigeminal motor neuropathy is an uncommon entity characterized by selective motor involvement of the mandibular division of the trigeminal nerve, most commonly presenting unilaterally. This case highlights the role of MRI in identifying characteristic chronic denervation changes, excluding secondary causes, and detecting subtle clinically occult contralateral abnormalities that may otherwise be overlooked. Early recognition of this rare neuropathy may help avoid unnecessary investigations and support appropriate clinical evaluation.

## Introduction

Pure trigeminal motor neuropathy (PTMN) is a rare cranial neuropathy characterized by selective involvement of the motor fibers of the mandibular division of the trigeminal nerve, resulting in weakness and atrophy of the muscles of mastication while preserving facial sensation [1,2]. Because the trigeminal nerve is predominantly sensory, isolated motor dysfunction is uncommon and may remain clinically underrecognized. Patients typically present with progressive facial asymmetry, jaw deviation, difficulty chewing, or painless wasting of the temporalis and masseter muscles. The indolent clinical course and absence of sensory deficits often lead to delayed diagnosis or misdiagnosis as temporomandibular joint dysfunction, primary myopathy, skull base pathology, or other causes of unilateral facial atrophy [3-5].

Magnetic resonance imaging plays an important role in diagnosis by demonstrating denervation-related muscular atrophy and excluding secondary causes such as skull base lesions, inflammatory processes, neoplasms, or brainstem pathology [6,7]. Chronic trigeminal motor denervation characteristically manifests as fatty replacement and volume loss involving the muscles of mastication, particularly the temporalis, masseter, and pterygoid muscles. High-resolution imaging of the trigeminal nerve pathway further assists in excluding compressive or infiltrative abnormalities affecting the cisternal segment or extracranial course of the mandibular division [8-10].

Most reported cases of PTMN describe unilateral involvement [1-7]. We present a case of predominantly left-sided PTMN with subtle contralateral MRI abnormalities suggesting possible early subclinical involvement, highlighting the role of MRI in detecting early denervation-related changes and excluding secondary causes.

## Case presentation

A 54-year-old woman presented with progressive left-sided facial asymmetry and jaw weakness over a period of three years. The symptoms developed insidiously and were associated with increasing difficulty during mastication. There was no history of facial pain, numbness, paresthesia, trauma, prior surgery, infection, constitutional symptoms, or systemic neurological illness. No visual disturbance, dysphagia, hearing loss, or weakness involving other cranial nerve distributions was reported. No right-sided facial symptoms or masticatory complaints were noted clinically.

Clinical examination revealed noticeable wasting over the left temporal and mandibular regions, producing facial asymmetry. Mild weakness of mastication was present, with reduced bulk of the left masseter and temporalis muscles. No definite right-sided masticatory weakness was identified clinically. Facial sensation across all divisions of the trigeminal nerve was preserved. The remaining cranial nerves were intact, with no evidence of facial nerve dysfunction, bulbar weakness, ocular motility abnormality, or generalized neuromuscular disorder.

**Figure 1:**
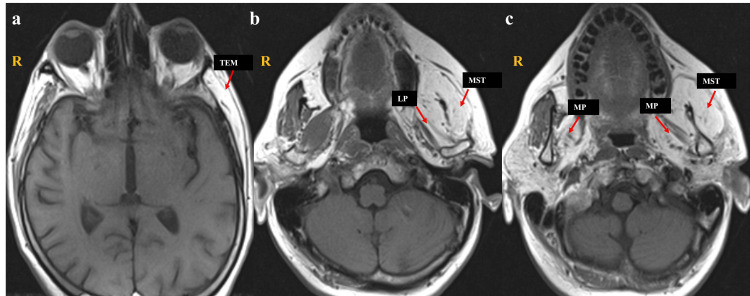
Axial T1-Weighted MRI Demonstrating Chronic Denervation-Related Fatty Atrophy of the Muscles of Mastication Axial T1-weighted non-fat-saturated MR images obtained at the level of the temporalis (a), lateral pterygoid (b), and medial pterygoid (c) muscles demonstrate marked volume loss and near-complete fatty replacement involving the left temporalis (TEM), masseter (MST), lateral pterygoid (LP), and medial pterygoid (MP) muscles, consistent with chronic denervation changes. Fatty infiltration and mild volume loss are also noted within the right medial pterygoid muscle. R = right side.

**Figure 2:**
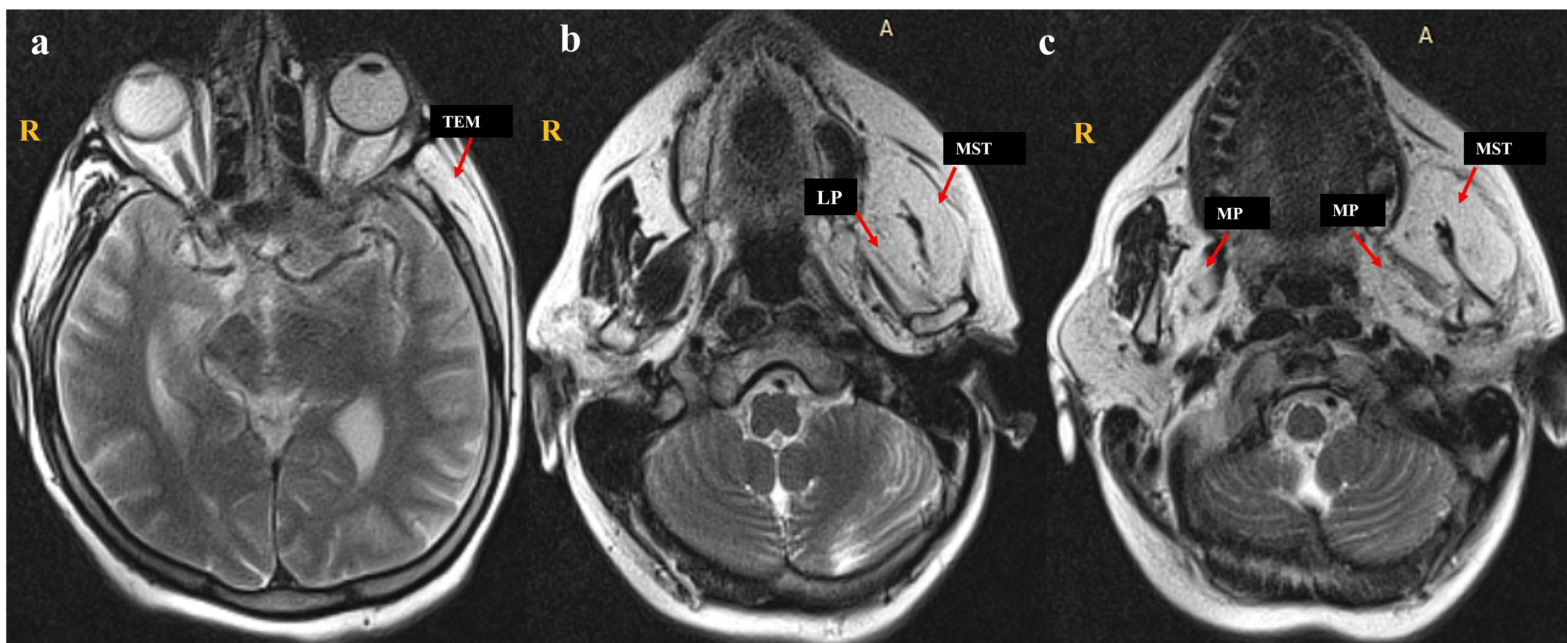
Axial T2-Weighted MRI Demonstrating Chronic Denervation Changes in the Muscles of Mastication Axial T2-weighted non-fat-saturated MR images obtained at the level of the temporalis (a), lateral pterygoid (b), and medial pterygoid (c) muscles demonstrate persistent hyperintense fatty signal alteration with marked volume loss involving the left temporalis (TEM), masseter (MST), lateral pterygoid (LP), and medial pterygoid (MP) muscles, consistent with chronic denervation changes. Fatty infiltration and mild volume loss are again noted within the right medial pterygoid muscle. R = right side.

**Figure 3:**
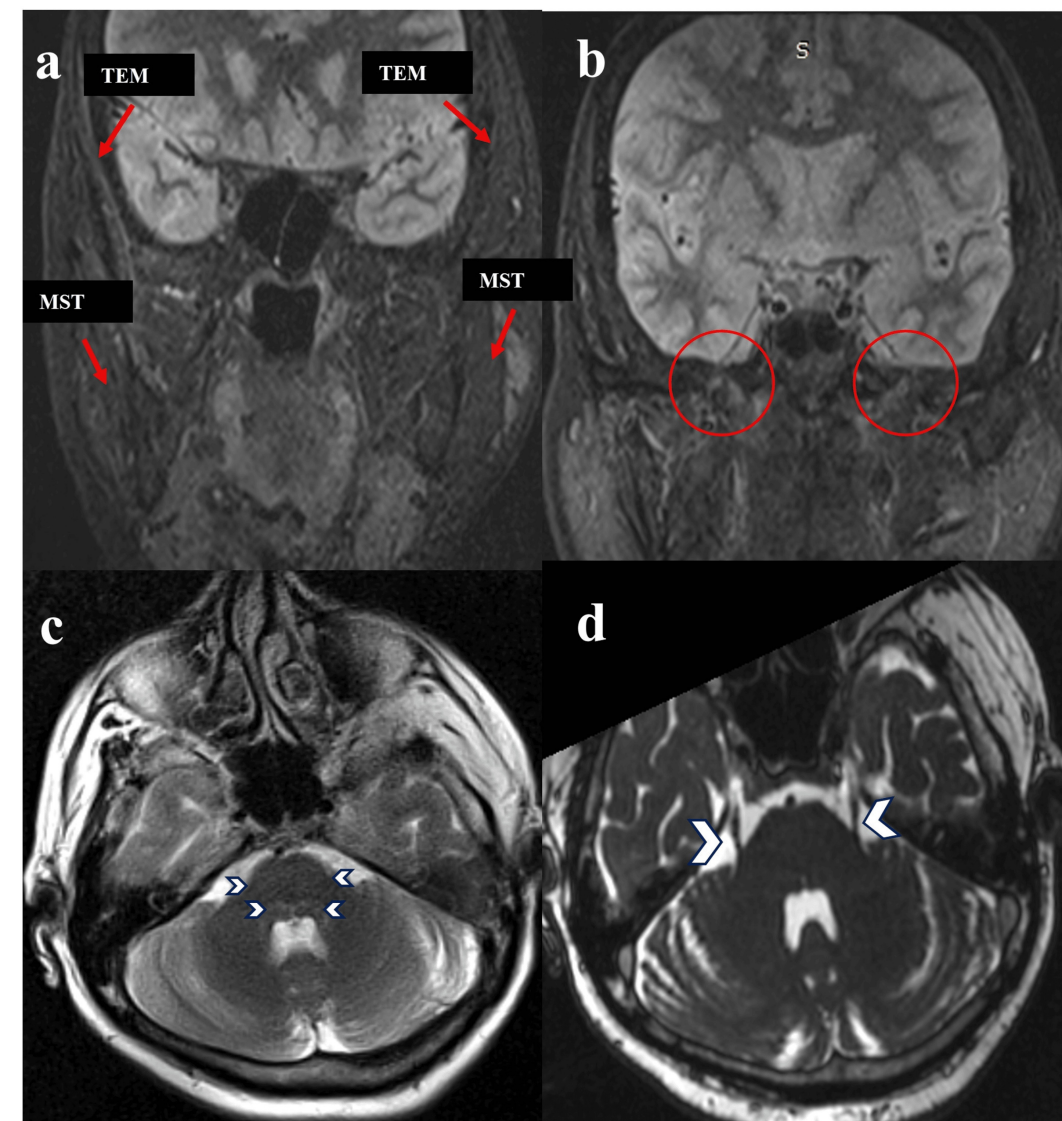
Fat-Suppressed and High-Resolution MRI Evaluation of the Trigeminal Nerve Pathway Coronal STIR images (a) demonstrate marked fatty atrophy of the left temporalis (TEM) and masseter (MST) muscles without associated muscle edema, supporting chronic denervation changes. The coronal STIR image through the skull base (b) demonstrates no abnormal soft tissue lesion or abnormal signal intensity along the expected foraminal course of the mandibular division of the trigeminal nerve bilaterally (circles). Axial T2-weighted image at the pontine level (c) demonstrates no focal signal abnormality within the pontine tegmentum. High-resolution 3D constructive interference in steady state (3D-CISS) image (d) shows symmetrical cisternal segments of both trigeminal nerves (arrowheads) without thickening, compression, or signal abnormality.

Magnetic resonance imaging of the face demonstrated marked fatty atrophy involving the left temporalis, masseter, medial pterygoid, and lateral pterygoid muscles, characterized by significant volume loss and fatty replacement. Axial T1- and T2-weighted non-fat-saturated images demonstrated near-complete fatty replacement of the left-sided muscles of mastication with associated volume loss (Figures 1, 2). Coronal STIR/fat-suppressed images showed no associated muscle edema, supporting chronic denervation changes (Figure 3A). No obvious thickening or abnormal STIR hyperintensity was identified along the expected course of the trigeminal nerve at the foramen ovale (Figure 3B). Fatty infiltration and mild volume loss were also identified within the right medial pterygoid muscle, suggesting subtle early subclinical contralateral involvement (Figures 1C, 2C).

T2-weighted images through the pontine level demonstrated no focal brainstem abnormality, while high-resolution 3D constructive interference in steady state (3D-CISS) sequences showed symmetrical and normal cisternal segments of both trigeminal nerves without thickening, compression, or signal abnormality (Figures 3C, 3D).

Electromyography demonstrated chronic neurogenic denervation involving the left masseter and temporalis muscles without evidence of generalized neuropathy or primary myopathy. Detailed right-sided electromyography findings were not available. The subtle right-sided imaging findings were therefore considered subclinical and MRI-based. Laboratory evaluation, including inflammatory, autoimmune, and infectious markers, revealed no abnormality suggestive of secondary neuropathic etiology.

Considering the clinical, imaging, and electrophysiologic findings, a diagnosis of left-sided pure trigeminal motor neuropathy with subtle MRI-based contralateral changes suggesting early subclinical involvement was considered.

## Discussion

The present case demonstrated marked chronic denervation changes involving the left muscles of mastication with subtle contralateral MRI abnormalities in the right medial pterygoid muscle, suggesting possible early subclinical involvement. Clinically and electrophysiologically, the disease remained predominantly left-sided, highlighting the potential role of MRI in detecting subtle clinically occult contralateral changes.

The trigeminal motor nucleus lies within the pontine tegmentum, and its efferent fibers travel with the mandibular division of the trigeminal nerve to innervate the temporalis, masseter, medial pterygoid, and lateral pterygoid muscles. Selective injury to these fibers may occur without sensory involvement, suggesting preferential motor pathway dysfunction. Although the precise pathogenesis remains uncertain, several mechanisms have been proposed, including viral neuritis, autoimmune-mediated inflammation, trauma, microvascular ischemia, and idiopathic degeneration [1,6,7]. In many reported cases, including the present case, no definite precipitating factor is identified despite clinical and laboratory evaluation.

Most reported cases of PTMN demonstrate unilateral involvement [1-5]. Contralateral MRI abnormalities in PTMN remain distinctly uncommon and sparsely documented in the literature. The present case is unusual because of predominant left-sided clinical and electrophysiologic involvement with subtle contralateral MRI abnormalities involving the right medial pterygoid muscle. Such findings may represent early subclinical extension of disease or an evolving neuropathic process. Recognition of subtle contralateral abnormalities is important because mild early denervation changes may be overlooked when clinical symptoms are predominantly unilateral.

Previously reported cases of pure trigeminal motor neuropathy described in the literature are summarized in Table 1, highlighting the predominance of unilateral disease and the rarity of reported asymmetric contralateral involvement.

**Table 1:** Previously Reported Cases of Pure Trigeminal Motor Neuropathy Summary of previously reported cases of pure trigeminal motor neuropathy (PTMN), including patient demographics, laterality, and proposed etiology. Most reported cases demonstrate unilateral involvement, while bilateral asymmetric disease remains exceedingly uncommon.

Author (Reference)	Year	Age/Sex	Laterality	Principal Imaging Finding	Etiology
Chiba et al. [6]	1990	57/M	Unilateral (Right)	Masticatory muscle atrophy	Idiopathic
Kang et al. [1]	2000	38/M	Unilateral (Right)	Denervation-related masticatory muscle atrophy	Idiopathic
Park et al. [8]	2006	63/F	Unilateral (Left)	Foramen ovale lesion affecting trigeminal motor branch	Secondary compressive (Neoplastic)
Chiba & Echigo [2]	2012	70/F	Unilateral (Left)	Masticatory muscle and mandibular ramus atrophy	Idiopathic
Wilson et al. [4]	2016	29/F	Unilateral (Left)	Focal masticatory muscle wasting	Idiopathic
Kämppi et al. [9]	2018	57/M	Unilateral (Left)	MRI evidence of chronic denervation	Idiopathic/ Borrelia
Nagure et al. [3]	2022	45/M	Unilateral (Right)	Fatty atrophy of masticatory muscles	Autoimmune (post-viral)
Kinugawa et al. [5]	2022	83/M	Unilateral (Right)	Chronic unilateral denervation changes	Idiopathic
Present Case	2026	54/F	Bilateral asymmetric (Left >> Right)	Extensive unilateral fatty atrophy with subtle contralateral involvement	Idiopathic

Magnetic resonance imaging plays a central role in diagnosis by demonstrating denervation-related muscular changes and excluding secondary pathology [3,5,10]. In chronic denervation, affected muscles demonstrate progressive volume loss and fatty replacement, typically appearing hyperintense on T1-weighted images due to fatty infiltration. T2-weighted sequences may also demonstrate chronic fatty signal alteration depending on the stage of denervation, while fat-suppressed sequences help exclude active muscle edema. High-resolution MRI sequences, including 3D constructive interference in steady state (3D-CISS) imaging, additionally permit evaluation of the cisternal trigeminal nerve segments and adjacent skull base regions for compressive or infiltrative pathology. MRI, therefore, provides a comprehensive assessment of the entire trigeminal pathway, including the brainstem, cisternal segment, skull base foramina, extracranial nerve course, and muscles of mastication.

Electromyography serves as an adjunctive investigation by confirming chronic neurogenic denervation and distinguishing neuropathic atrophy from primary myopathic processes [1,5]. Correlation between MRI findings and electrophysiologic abnormalities improves diagnostic confidence, particularly in cases lacking structural abnormalities along the trigeminal nerve course. Because PTMN remains a diagnosis of exclusion, recognition of characteristic imaging findings is important to avoid delayed diagnosis and unnecessary investigations. The combination of isolated trigeminal motor dysfunction, preserved sensory examination, chronic denervation changes within the muscles of mastication, and absence of structural pathology strongly supports the diagnosis. This case contributes to the limited literature describing subtle contralateral MRI abnormalities in PTMN and highlights the value of high-resolution MRI in identifying clinically occult denervation changes.

The differential diagnosis of unilateral or asymmetric masticatory muscle atrophy is broad and includes temporomandibular joint dysfunction, chronic disuse atrophy, infiltrative skull base lesions, perineural tumor spread, progressive hemifacial atrophy, motor neuron disease, and primary inflammatory or dystrophic myopathies [4,8,9]. Temporomandibular disorders may cause pain and altered mastication, but do not typically produce selective denervation of trigeminal-innervated muscles. Progressive hemifacial atrophy usually involves cutaneous, subcutaneous, and osseous facial structures rather than isolated masticatory muscle denervation. Myopathic disorders tend to produce diffuse muscular involvement rather than a cranial nerve distribution pattern. Careful imaging assessment is therefore essential to narrow the differential diagnosis and establish a neuropathic etiology.

Because PTMN remains a diagnosis of exclusion, recognition of characteristic imaging findings is important to avoid delayed diagnosis and unnecessary investigations. The combination of isolated trigeminal motor dysfunction, preserved sensory examination, chronic denervation changes within the muscles of mastication, and absence of structural pathology strongly supported the diagnosis in the present case. This case highlights the value of high-resolution MRI in identifying subtle clinically occult contralateral abnormalities that may suggest early subclinical involvement.

## Conclusions

Pure trigeminal motor neuropathy is a rare and often underrecognized cranial neuropathy that should be considered in patients presenting with chronic facial asymmetry, jaw weakness, and isolated atrophy of the muscles of mastication in the absence of sensory deficits. MRI plays an important role in establishing the diagnosis by demonstrating characteristic chronic denervation-related fatty atrophy and excluding secondary structural causes along the trigeminal nerve pathway. In the present case, subtle contralateral MRI abnormalities were identified despite predominantly left-sided clinical and electrophysiologic involvement, suggesting possible early subclinical changes. Recognition of such imaging findings, together with appropriate clinical and electrophysiologic correlation, may help support diagnosis and avoid unnecessary investigations. This case further highlights the importance of comprehensive MRI evaluation in rare trigeminal motor neuropathies.
